# *CDKN1A* and *FANCD2* are potential oncotargets in Burkitt lymphoma and multiple myeloma

**DOI:** 10.1186/s40164-015-0005-2

**Published:** 2015-03-27

**Authors:** Seong-Su Han, Van S Tompkins, Dong-Ju Son, Sangwoo Han, Hwakyung Yun, Natalie L Kamberos, Casey L Dehoedt, Chunyan Gu, Carol Holman, Guido Tricot, Fenghuang Zhan, Siegfried Janz

**Affiliations:** Department of Pediatrics, University of Iowa Carver College of Medicine, Iowa City, IA USA; Department of Pathology, University of Iowa Carver College of Medicine, Iowa City, IA USA; Department of Health and Human Physiology, University of Iowa Carver College of Medicine, Iowa City, IA USA; Department of Internal Medicine, University of Iowa Carver College of Medicine, Iowa City, IA USA; School of Applied Biosciences, Kyungpook National University, Daegu, 702-701 South Korea; Department of Biological Sciences, Hanseo University, Choognam, South Korea

**Keywords:** p21 tumor suppressor, Fanconi anemia and breast cancer DNA damage repair, Genetically engineered mouse models of human cancer, Molecularly targeted cancer therapy

## Abstract

**Background:**

Comparative genetic and biological studies on malignant tumor counterparts in human beings and laboratory mice may be powerful gene discovery tools for blood cancers, including neoplasms of mature B-lymphocytes and plasma cells such as Burkitt lymphoma (BL) and multiple myeloma (MM).

**Methods:**

We used EMSA to detect constitutive NF-κB/STAT3 activity in BL- and MM-like neoplasms that spontaneously developed in single-transgenic IL6 (interleukin-6) or MYC (c-Myc) mice, or in double-transgenic IL6MYC mice. qPCR measurements and analysis of clinical BL and MM datasets were employed to validate candidate NF-κB/STAT3 target genes*.*

**Results:**

qPCR demonstrated that IL6- and/or MYC-dependent neoplasms in mice invariably contain elevated mRNA levels of the NF-κB target genes, *Cdkn1a* and *Fancd2*. Clinical studies on human *CDKN1A*, which encodes the cell cycle inhibitor and tumor suppressor p21, revealed that high p21 message predicts poor therapy response and survival in BL patients. Similarly, up-regulation of *FANCD2*, which encodes a key member of the Fanconi anemia and breast cancer pathway of DNA repair, was associated with poor outcome of patients with MM, particularly those with high-risk disease.

**Conclusions:**

Our findings suggest that *CDKN1A* and *FANCD2* are potential oncotargets in BL and MM, respectively. Additionally, the IL-6- and/or MYC-driven mouse models of human BL and MM used in this study may lend themselves to the biological validation of *CDKN1A* and *FANCD2* as molecular targets for new approaches to cancer therapy and prevention.

**Electronic supplementary material:**

The online version of this article (doi:10.1186/s40164-015-0005-2) contains supplementary material, which is available to authorized users.

## Background

Comparative histopathologic, genomic and biological analyses of malignant tumor counterparts in humans and mice afford a powerful approach to improve our understanding of evolutionarily conserved signaling networks that underlie oncogenesis and are thus of great significance for public health. An important objective of cross-species analysis of neoplastic development is the discovery of concordantly deregulated genes that play an important role in tumor development and progression, response to therapy, acquisition of drug resistance, and clinical outcome. Protein-encoding genes that are overexpressed in cancer cells and potentially inhibitable by small compounds are of particular interest to that end, because they are actionable in terms of molecularly targeted drug development. Here, we take advantage of MYC- [1], IL6- [2] and IL6MYC-transgenic (Tg) mice [3,4] that recapitulate important features of human Burkitt lymphoma (BL) or multiple myeloma (MM) to uncover up-regulated candidate cancer genes that might have been overlooked in other studies. We show that MYC and/or IL-6-driven B cell and plasma cell tumors of mice exhibit constitutive NF-κB activity that leads to overexpression of NF-κB target genes such as *Cdkn1a* and *Fancd2*. These genes encode the well-established tumor suppressor, p21, and a key member of the Fanconi anemia/breast cancer DNA damage repair pathway, respectively. Interrogation of a well-annotated clinical dataset (n = 351) suggested that *FANCD2* is a MM gene. Likewise, *in vitro* studies on tumor cell lines and clinical outcome results indicated that *CDKN1A* may be a BL oncogene. Although additional work is warranted before the utility of CDKN1A and FANCD2 as molecular targets for drug development can be fully evaluated, our study underlines the value of comparative oncogenomic and molecular genetic research on human-mouse cancer counterparts for developing new approaches to cancer therapy and prevention.

## Results

### Constitutive NF-κB and STAT3 activity in IL-6 and/or MYC-driven B cell and plasma cell tumors in mice

**Figure 1:**
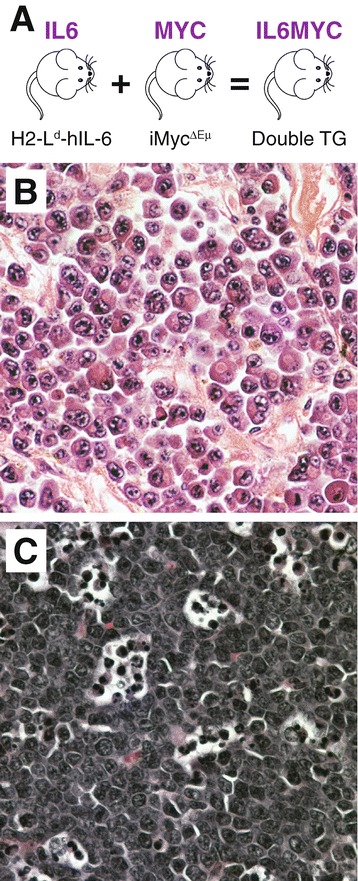
**MYC and/or IL-6 driven mouse models of human Burkitt lymphoma (BL) and multiple myeloma (MM). (A)** Overview of transgenic (Tg) mice used in this study. IL6 mice harbor a widely expressed human interleukin-6 transgene designated H2-L^d^-hIL6 [40,41]. MYC mice contain a mouse Myc (c-myc) cDNA transgene that has been inserted in the immunoglobulin heavy-chain locus with the help of gene targeting in embryonic stem cells [42]. IL6MYC mice were generated by intercrossing homozygous-Tg MYC mice and heterozygous-Tg IL6 mice, followed by selection of double-Tg offspring. All mice were on the genetic background of BALB/c **(C)**. **(B)** Photomicrograph of a representative histologic section of the type of myeloma-like plasma cell tumor that arises consistently in both IL6 and IL6MYC mice (H&E, original magnification 40x). Compared to IL6 mice, tumor onset is shorter and tumor incidence is higher in IL6MYC mice. **(C)** Representative tissue section of the main type of tumors observed in MYC mice: Burkitt-like lymphoblastic B-cell lymphoma that exhibits the typical “starry sky” morphology due to tingible body macrophages that engulf apoptotic tumor cells (H&E, original magnification 40x).

**Figure 2:**
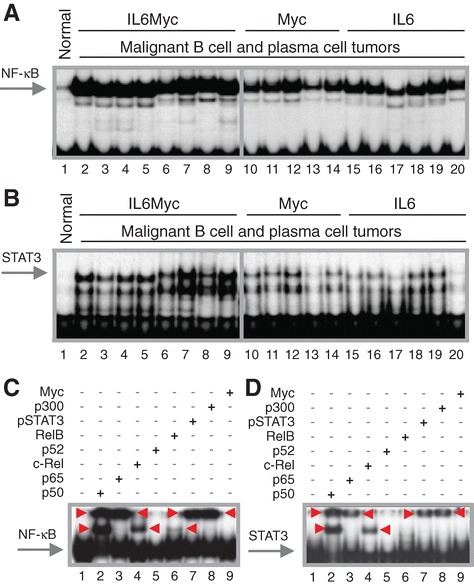
**Constitutive activation of NF-κB/Stat3 signaling in malignant B cells and plasma cells of MYC- and/or IL-6-transgenic mice. (A)** EMSA result indicating high levels of NF-κB DNA-binding activity in BL-like lymphomas and MM-like plasma cell tumors that developed in double-transgenic IL6MYC mice (lanes 2–9) or single-transgenic MYC (lanes 10–14) or IL6 mice (lanes 15–20). Normal B cells (lane 1) were included as control. **(B)** EMSA result demonstrating high levels of Stat3DNA-binding activity in the same samples used in panel A. **(C)** EMSA super-shifts indicating both involvement of p50, p65, c-Rel and, to a lesser extent, RelB in NF-κB activation and physical association of NF-κB proteins with Stat3 and p300. Red arrowheads denote shifted bands. In lanes 2–8, NE (10 μg) was incubated with 2 μg of one of the Abs to NF-κB, pStat3 or p300 indicated above the gel image. Antibody to Myc (lane 9) or omission of Ab (lane 1) were used as controls. Red arrowheads denote shifts. **(D)** EMSA super-shifts suggesting physical association of NF-κB with pStat3 and p300. Red arrowheads, shifted bands; lanes 2–8, Abs to NF-κB, pStat3 or p300; lanes 1 and 9, controls.

Because NF-κB and STAT3 are important regulators of B-lineage neoplasms in humans and mice, we used EMSA to examine NF-κB and STAT3 activity in MACS-purified B220^+^ samples from 19 primary tumors obtained from single-transgenic IL-6 (n = 6) or MYC (n = 5) mice and double-transgenic IL6MYC (n = 8) mice (Figure 1). Compared to normal B220^+^ splenocytes used as control, all tumor samples contained a significantly elevated DNA-binding activity of both transcription factors (Figure 2A and B). To ascertain the subunit composition of NF-κB dimers, we performed super-shift assays using nuclear extracts (NEs) from tumor cells isolated from enlarged spleens of IL6MYC mice. We included antibodies (Abs) to all 5 NF-κB subunits (Figure 2C) and observed notable shifts for three: p50 (lane 2), p65 (lane 3) and c-Rel (lane 4). Ab to RelB caused only a faint shift (lane 6) and p52 seemed to be uninvolved (lane 5). Similar to our previous findings on the MYC-dependent mouse lymphoma cell line iMyc^Eμ^ [5], Ab to p50 shifted two NF-κB-specific bands to higher molecular-weight positions on EMSA gels, whereas Ab to p65 or c-Rel shifted only the upper or lower band, respectively. Both activated STAT3, pSTA3, and acetyltransferase p300, a positive regulator of NF-κB/STAT3 signaling [6,7], were physically associated with NF-κB in IL6MYC tumor cells (lanes 7 and 8). Next, we confirmed these findings with the assistance of STAT3 super-shift assays, using the same NEs as in panel C (Figure 2D). We found that three NF-κB subunits (p50, lane 2; p65, lane 3; c-Rel, lane 4), pStat3 (lane 7) and p300 (lane 8) participated in protein complexes that were able to bind a canonical STAT3 DNA recognition sequence. The results presented in Figure 2 suggested that the constitutive NF-κB activity in IL6MYC tumors is comprised of both p50 homo and p50 hetero (with p65 and c-Rel) dimers that are further associated with p300 and pSTAT3. While the association with the former has not yet been reported to the best of our knowledge, the latter finding is in agreement with reports on the physical association of STAT3 and NF-κB in various cell types [8] including MYC-transgenic B-lymphocytes [5].

### Up-regulation of *Cdkn1a* and *Fancd2* in mouse tumor cells

**Figure 3:**
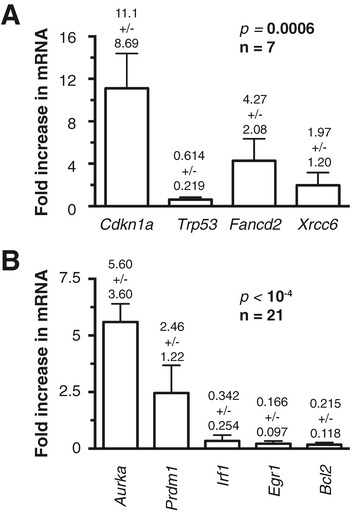
**Gene expression changes in malignant B cells and plasma cells of MYC- and/or IL-6-transgenic mice.** Shown are mean values and standard deviations of qPCR results obtained in triplicate. The results of Kruskal-Wallis comparisons of median gene expression values are indicated to the upper right. **(A)** *Cdkn1a* (cyclin-dependent kinase inhibitor 1A) and *Trp53* (transformation related protein 53) encode the tumor suppressors p21 and p53, respectively. *Fancd2*, Fanconi anemia, complementation group D2; *Xrcc6*, complementation X-ray repair complementing defective repair in Chinese hamster cells 6. **(B)** *Aurka*, aurora kinase A; *Prdm1*, PR domain containing 1, with ZNF domain (aka Blimp-1); *Irf1*, interferon regulatory factor 1; *Egr1*, early growth response 1; *Bcl2*, B cell leukemia / lymphoma 2. Wild-type status of *Cdkn1a/p21* sequence was confirmed by DNA sequencing in eight tumors from C.IL6/iMyc^∆Eμ^ mice (data not shown).

qPCR analysis of RNA from tumor cells isolated from seven different IL-6- and/or MYC-induced neoplasms included in Figure 2A and B demonstrated that three well-established NF-κB target genes were consistently up-regulated in this sample: *Cdkn1a*, a target of the tumor-suppressor p53, and two DNA repair genes, *Fancd2* and *Xcrcc6* (Figure 3A). Mean *Cdkn1a* message in tumor cells was 11.1-fold elevated compared to normal B cells (standard deviation 8.69; *p* = 0.0169 by Mann–Whitney), whereas expression of *Trp53* (encodes p53) was down regulated (0.614 ± 0.219; *p* = 0.0006). *Fancd2* and *Xcrcc6* expression was increased by a factor of 4.27 ± 2.08 (*p* = 0.0006) and 1.97 ± 1.20 (*p* = 0.0169), respectively. Median expression values of the 4 genes varied significantly using Kruskall-Wallis test (*p* = 0.0006). Because the small amounts of tumor RNA available were insufficient to evaluate additional genes of interest, we prepared RNA from another tumor set (n = 21) obtained from an independent cohort of IL6MYC mice. Continuing with the qPCR analysis, we first confirmed the changes presented in Figure 3A (results not shown) and then showed that the genes encoding aurora kinase A (*Aurka*) and the germinal center B cell master transcription factor Blimp-1 (*Prdm1*) were up-regulated in tumor cells, whereas expression of *Irf1* (interferon regulatory factor 1, *Egr1* (early growth response 1) and *Bcl2* (B cell leukemia 2) was decreased (Figure 3B). The differences in gene expression were highly significant for each gene individually (compared to normal B cells; *p* < 0.0001, Mann Whitney) and considered as a set (*p* < 0.0001, Kruskall-Wallis). *Cdkn1a*, *Fancd2*, *Xcrcc6*, *Aurka* and *Prdm1* are drug-inhibitable overexpressed protein-encoding genes that might be of interest for the development of molecularly targeted interventions.

### CDKN1A is a candidate oncogene in human Burkitt lymphoma (BL)

**Figure 4:**
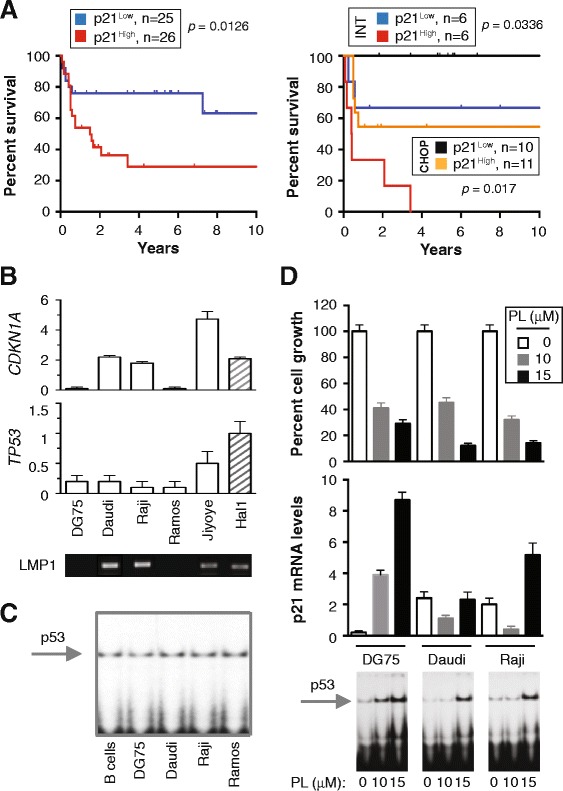
**CDKN1A expression prognosticates poor outcome in human BL. (A)** Kaplan-Meier curves indicating decreased survival of BL patients expressing high levels of p21 message (Mantel-Cox log-rank analysis). Data are from Dave et al. (GSE4732, probe 202284) [15]. The left panel depicts survival of 51 patients evenly split according to p21 expression into a p21^Low^ group and a p21^High^ group. The former demonstrated better outcome (*p* = 0.0126). The right panel shows survival of 33 patients treated with either a CHOP-like regimen or an intensive (INT) drug regimen supplemented in some cases with autologous hematopoietic stem cell transplantation. p21^Low^ patients demonstrated better outcomes than p21^High^ patients in both treatment arms. **(B)** *CDKN1A* and *TP53* expression in 5 human BL cell lines and 1 mouse BL-like cell line, Hal1, derived from a LMP1-transgenic lymphoma. Daudi, Raji and Jiyoye are EBV^+^ and thus express virus-encoded *LMP1*. DG75 and Ramos are EBV^-^. Because LMP1 activates NF-κB in malignant B cells [43], it is possible that p21 expression in LMP1^+^ cells is driven, in part, by LMP1. **(C)** EMSA indicating p53 DNA-binding activity in BL and normal B cells. **(D)** Piperlongumin (PL)-induced activation of the p53-p21 stress response in BL cells. Shown at the top is the growth inhibition of cells upon treatment with 10 μM or 15 μM PL for 24 hrs (MTS assay). Mean values and error bars, which represent the standard deviation from triplicate experiments, are plotted. The center panel shows the corresponding p21 message levels (qPCR). Differences in both panels were not significant (Mann–Whitney, *p* = 0.1). Presented at the bottom are the corresponding EMSA results at 24 hrs, indicating that the drug dose-dependent induction of p53 DNA-binding activity was more vigorous at 10 μM PL in EBV^−^ DG75 cells compared to EBV^+^ Daudi and Raji cells.

*Cdkn1a* was of special interest to us because (1) evidence has emerged that p21 may promote tumor development despite its more widely known role as cell cycle inhibitor and tumor suppressor [9], (2) the p21 and p53 message levels presented in Figure 3A were in line with findings in oral squamous cell carcinoma [10], head and neck cancer [11] and breast cancer [12] indicating that expression of p21 may be independent of p53, (3) mCD40-LMP1/iMyc^Eμ^-driven BL-like neoplasms in mice contain elevated levels of p21 mRNA [13] and (4) findings indicate that RelB, which is involved in IL6MYC-dependent NF-κB activation as shown in Figure 2C, may be a positive regulator of p21’s oncogenic function [14]. To follow up on the possibility that p21 has oncogenic properties in neoplasms of mature B-lymphocytes, and to evaluate the clinical significance, if any, of up-regulation of *CDKN1A*, we interrogated publicly available gene expression profiles of human non-Hodgkin lymphomas and MM. The dataset of Dave et al. [15] (GSE4732) was most helpful because (1) it permitted us to examine overall survival as a function of *CDKN1A* expression, (2) it included only cases of BL that had been validated using molecular signatures and (3) survival data was available for a sufficient number of patients (n = 51). We stratified the patients into 2 groups according to high and low *CDKN1A* expression at diagnosis (using the median level of p21 as cut-off), generated a Kaplan-Meier plot (Figure 4A, left) and found that p21^High^ patients had a significantly worse outcome (*p* = 0.0126) compared with their p21^Low^ counterparts. Next, we determined whether the same held true when patients were first stratified according to treatment, which was reported for 33 of 51 (65%) cases. Indeed, p21^High^ patients receiving either a CHOP-like or even more intensive treatment regimen (INT) fared worse than the p21^Low^ group ((Figure 4A, right). Unlike BL, survival of p21 expression-stratified DLBCL patients in the Dave et al. study was not different (Additional file 1: Figure S1). These results are consistent with an oncogenic role for p21 in BL and suggest that p21 expression is a marker of high-risk disease.

### Induction of *CDKN1A* in the course of a drug-induced stress response in BL cells

The availability of representative tumor cell lines afforded an opportunity to examine the cancer cell-autonomous properties of p21 in human BL in greater depth. We selected 5 independent cell lines, 3 of which are infected with Epstein Barr virus (EBV) and thus express the virus-encoded oncoprotein LMP1: Daudi, Raji and Jiyoye. Two cell lines, DG75 and Ramos, do not harbor EBV (Figure 4B, bottom). Mouse Hal2G1 tumor cells, which carry the mCD40-LMP1 and iMyc^Eμ^ transgenes [13], were included for comparison. EBV^+^ BL cells contained increased *CDKN1A* mRNA compared to EBV^−^ BL cells (Figure 4B, top), whereas *TP53* message (Figure 4B, center) and p53 DNA-binding activity (Figure 4C) were consistently low irrespective of EBV/LMP1 status. Next we measured *CDKN1A* expression under conditions of drug-induced stress imposed by treatment of cells with the cancer-inhibiting agent, piperlongumine (PL). In agreement with our previous finding that PL kills human BL and mouse BL-like cells by virtue of a mechanism that includes inhibition of NF-κB [13,16], we found that PL kills 3 of 3 BL lines with similar efficacy (Figure 4D, top). PL-dependent induction of both *CDKN1A* (Figure 4D, center) and p53 DNA binding activity (Figure 4D, bottom) was more robust in EBV^−^ DG75 cells than in EBV^+^ Daudi and Raji cells, but a larger study is warranted to decide whether this is truly associated with EBV/LMP1 status or caused by coincidence. Be this as it may, the results described above implicate the activation of the p53/p21 pathway in the PL-dependent stress response in BL cells.

### *FANCD2* is a candidate multiple myeloma (MM) gene

**Figure 5:**
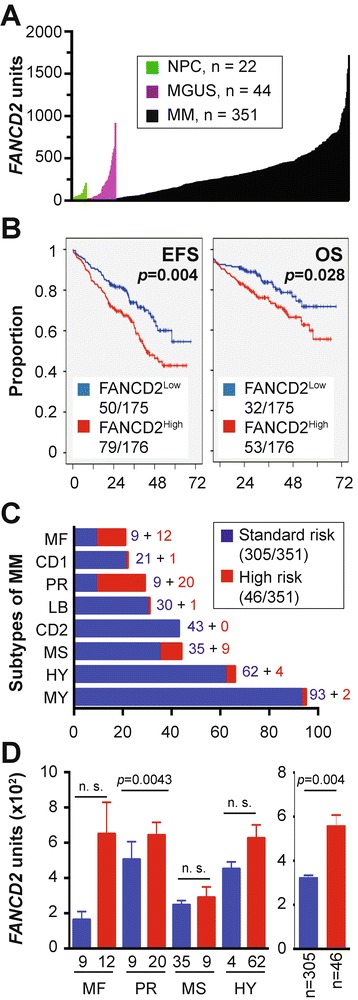
***FANCD2*** **expression predicts poor survival in a subset of patients with newly diagnosed multiple myeloma. (A)** *FANCD2* mRNA levels (gene probe ID 242560) in normal bone marrow (BM) plasma cells (NPC, green), “premalignant” BM plasma cells from individuals with monoclonal gammopathy of undetermined significance (MGUS, purple) or malignant plasma cells from patients with multiple myeloma (MM, black). **(B)** Reduced event-free survival (EFS) and overall survival (OS) in myeloma patients with elevated *FANCD2* levels (log-rank analysis). Of 351 patients, 175 and 176 patients were arbitrarily categorized as “Low FANCD2” and “High FANCD2,” respectively, using the median *FANCD2* level in this cohort as cut-off. EFS and OS data were available from 129 (37%) and 85 (24%) patients, respectively. **(C)** Proportion of myelomas (n = 351) that fell into 8 different subgroups of the disease based on cytogenetic features (e.g., ploidy and chromosomal translocations) and molecular genetic features (e.g., gene expression signatures). From top to bottom, the following subgroups are distinguished: MF, MAF/MAFB; CD1, CCND1/CCND3 group 1; PR, proliferation; LB, low bone disease; CD2, CCND1/CCND3 group 2; MS, MMSET; HY, hyperdiploid; MY, myeloid [39]. The distribution of standard-risk (blue) and high-risk cases (red) according to the 70-gene signature [18] is also indicated. **(D)** Shown to the left is elevation of *FANCD2* message in high-risk myeloma (red) vs. standard-risk myeloma (blue) in 4 of 8 subgroups of the disease included in panel C. Mean values (microarray units) and standard error of the mean (SEM) are plotted. Mann–Whitney tests were used for statistical analyses (n. s., not significant). Shown to the right is the increase in *FANCD2* mRNA in high-risk disease (red; 13%) vs. standard-risk disease (blue; 87%) in 351 myeloma patients. Mean values and SEM are plotted. Median *FANCD2* levels in high-risk and standard-risk disease were 473 and 269, respectively.

Next, we focused on another candidate gene included in Figure 3, *FANCD2*, because the analysis of a clinically annotated MM dataset for which Affymetrix-based gene expression results were available suggested that this gene may be important for myeloma. Figure 5A depicts the levels of *FANCD2* in 351 patients with newly diagnosed myeloma, according to increasing gene expression. The mean *FANCD2* mRNA level in myeloma cells (351 ± 268, n = 351) was 4.3-fold higher than in normal plasma cells (NPCs) from the bone marrow (81.2 ± 54.1, n = 22) and 1.8-fold higher than in plasma cells from individuals with monoclonal gammopathy of undetermined significance (MGUS; 195 ± 207, n = 44). Both increases were highly significant using Mann–Whitney analysis (*p* < 10^−4^). To test the possibility that *FANCD2* expression is of prognostic significance in myeloma, we used the median *FANCD2* level in myeloma cells (291 array units) as a cutoff to determine event-free survival (EFS) and overall survival (OS) separately in FANCD2^Low^ patients (n = 175) versus FANCD2^High^ patients (n = 176). Both EFS (*p* = 0.004) and OS (*p* = 0.028) were significantly shorter in the FANCD2^High^ cohort using log-rank analysis (Figure 5B). These findings suggested that up-regulation of *FANCD2* leads to increased myeloma aggressiveness.

### *FANCD2* is associated with high-risk myeloma

Keeping in mind that the prognosis of myeloma depends in large measure on tumor genetics [17] and that risk stratification models of myeloma rely on genetic features, such as changes in gene expression according to the 70-gene model [18], to assign newly diagnosed cases to standard-risk groups (median OS >10 years) and high-risk groups (2–3 years) [19], we analyzed whether FANCD2^High^ status might be associated with high-risk disease as defined by the 70-gene signature [18]. Forty-six of 351 myeloma patients (13%) carried this signature (Figure 5C), with most of them falling into four subgroups of myeloma: proliferation (PR, n = 20), MAF/MAFB (MF, n = 12), MMSET (MS, n = 9) and hyper-diploid (HY, n = 4). In all four subgroups, mean *FANCD2* levels were elevated in high-risk relative to standard-risk disease, but statistical significance was only reached in one subgroup, PR (*p* = 0.0043, Mann–Whitney test (Figure 5D, left). When all 46 high-risk cases were pooled and the mean *FANCD2* expression level of that pool was compared to all 305 standard-risk cases, the up-regulation of *FANCD2* in high-risk myeloma was highly significant (*p* = 0.004; Figure 5D, right). This result suggested that *FANCD2* is a bona fide high-risk myeloma gene as defined by the 70-gene model.

## Discussion

The main finding of this study is the implication of *FANCD2* and *CDKN1A* in high-risk MM and BL, respectively. *CDKN1A* was first identified as an inhibitor of cell cycle progression and tumor development that is up regulated by wild-type p53 [20,21]. Nowadays *CDKN1A* is more broadly considered as a regulator of fundamental cell-fate decisions, such as proliferation, differentiation and senescence [22]. With respect to oncogenesis, the tumor suppressor function of *CDKN1A* is well established, yet there is also growing evidence for oncogenic properties of *CDKN1A*-encoded p21. For instance, p21 is overexpressed in mouse fibroblasts undergoing transformation induced by ionizing radiation [23]. The underlying mechanism is not known but likely includes protection from apoptosis, which is emerging as key for p21’s oncogenic functions [24]. In line with that, cytoplasmic p21, the accumulation of which is stimulated by AKT- or IKKβ-dependent pathways, suppresses programmed cell death [25]. Furthermore, NF-κB-dependent up-regulation of p21 inhibits apoptosis in cells damaged by doxorubicin [26] or UV irradiation [27]. Importantly, tumor development studies in laboratory mice have demonstrated that deletion of *Cdkn1a* inhibits lymphoma in Trp53-deficient [28], Atm-deficient [29] and normal mice [30], rather than promoting lymphoma as one might expect from the loss of a tumor suppressor gene. In further agreement with p21’s survival-enhancing activity, lymphomas arising in Cdkn1a-deficient mice demonstrate a high rate of apoptosis [31]. In human cancers, overexpression of *CDKN1A* is frequently seen in carcinoma (prostate, cervix, breast, ovary, skin), brain tumor (glioma) and hematological malignancy [25]. With regard to the latter, strong experimental evidence indicates that p21’s pro-survival function plays an important role in the natural history of human leukemia [32]. These findings support the result of this study suggesting that p21 promotes BL by functioning as an oncogene in the mature B-cell lineage.

The observed overexpression of *Fancd2* and *Xrcc6* in myeloma-like plasma cell tumors from IL6MYC mice established an interesting parallel to human MM, in which the Fanconi anemia/breast cancer (FA/BRCA) DNA damage repair pathway has been identified as an important effector mechanism of myeloma cell responses to replicative stress induced by DNA alkylating agents, such as the widely used myeloma drug melphalan [33]. FA/BRCA has also been implicated in the acquisition of drug including bortezomib resistance by myeloma cells [34]. FANCD2 is a key player in the FA/BRCA pathway and XRCC6 is a facilitator of DNA double-strand break repair. Both repair proteins are subject to regulation by NF-κB (e.g., p50/RelB dimers) [33,35,36] and both intersect functionally with p21. The latter is illustrated by reports that p21 can activate FA-BRCA-dependent [37] and XRCC6-dependent [38] DNA repair, although this has not yet been demonstrated for myeloma. In this study, we provided clinical evidence for a role of FANCD2 in myeloma. Unlike *XRCC6* expression, which was not associated with survival in the Total Therapy 2 (TT2) myeloma dataset used here (n = 351), the message levels of *FANCD2* were significantly correlated with event-free and overall survival of patients with myeloma. Moreover, overexpression of *FANCD2* appeared to be a feature of high-risk myeloma, a subset of the disease for which new therapeutic approaches are urgently needed. Patients with high-risk myeloma have extremely poor outcomes; as such, they define an unmet medical need. The median overall survival for patients with standard-risk myeloma is >10 years years, yet that for patients with high-risk disease is 2–3 years, despite the application of aggressive, risk-adapted therapies including tandem autologous stem-cell transplantation (ASCT) and new myeloma drugs [19]. The results presented here support the view that *FANCD2* may be a bone fide high-risk myeloma gene that is worthy for consideration as molecular target for new, targeted therapies of patients with MM.

## Conclusion

Starting with the observation that IL-6 and/or MYC-driven B cell and plasma cell tumors in BALB/c mice exhibit constitutive NF-κB/STAT3 activity that leads to up-regulation of NF-κB target genes, we found that two genes of this sort, *CDKN1A* and *FANCD2*, are important for human BL and human MM, respectively. It is possible that these genes drive neoplastic development in the mature B-cell lineage, but this has not been demonstrated here. Additional functional and mechanistic studies are warranted before it can be decided whether CDKN1A and FANCD2 provide viable molecular targets for new therapeutic approaches to BL and MM. MYC-driven BL-like tumors and IL6MYC-driven MM-like tumors in laboratory mice may lend themselves as experimental model systems to that end.

## Methods

### Transgenic mice, human BL cell lines, and normal B-lymphocytes

The generation and tumor phenotypes of single-transgenic MYC [1] or IL6 [2] mice and double-transgenic IL6MYC mice [3,4] have been previously reported. All transgenes were on the genetic background of BALB/c (C). Breeding, maintenance and handling of mice was conducted according to IACUC guidelines and approved under University of Iowa ACURF study protocol 1301010. Human Burkitt lymphoma (BL) cell lines, purchased from ATCC (Manassas, VA), were maintained in RPMI 1640 supplemented with 10% heat-inactivated fetal bovine serum, in a humidified 5% CO_2_ incubator at 37°C. Human B cells were isolated from peripheral blood of healthy individuals as previously described [16]. Normal B220^+^ B cells were fractionated from the spleen of inbred C mice, using MACS® CD45R magnetic beads columns from Miltenyi Biotec (Auburn, CA).

### Quantitative reverse-transcription PCR (qPCR)

Reverse transcription, performed on 1 μg of TRIzol (Sigma-Aldrich, St. Louis, MO)-extracted total RNA, was followed by cDNA synthesis using the AMV reverse transcriptase kit (Roche, Indianapolis, IN). qPCR was performed with the help of TaqMan Universal PCR Master Mix (Applied Biosystems, Carlsbad, CA), using primers and 6-carboxyfluorescein (6-FAM) / Black Hole (BHQ)-labelled probes to specific target genes (IDT, Coralville, IA). Sequences of probes are available upon request. The Applied Biosystems 7900 HT device was used for amplification and detection of PCR product. ABI SDS v 2.3 software (Applied Biosystems, Carlsbad, CA) was employed for analyzing results. The Ct value for each gene was normalized to the internal reference control, *HPRT1* for human genes or *Hprt* for mouse genes, and represented as fold gene expression change relative to gene expression in normal human or mouse B cells or, in case of drug studies, to vehicle-treated cells.

### Cell proliferation

Proliferation was determined using the Cell Titer 96® MTS/PMS assay (Promega, Madison, WI). Briefly, 1 x 10^5^ cells in 100 μl growth media were plated into 96-well plates (Costar, Cambridge, MA). After 20 hours, 20 μl of MTS/PMS solution was added per well. Four hours later, the absorbance at 490 nm was measured using a Multiskan Spectrum plate reader (Thermo Scientific, Hudson, NH).

### Preparation of nuclear and cytosolic extracts

Cells (1 × 10^7^) were lysed with 400 μl of buffer A (10 mM KCl, 0.2 mM EDTA, 1.5 mM MgCl_2_, 0.5 mM DTT, and 0.2 mM PMSF) at 4°C for 10 minutes. Lysate was centrifuged for 5 minutes at 14,000 *g* and supernatants were collected as cytosolic extracts. Pellet was re-suspended in 100 μl ice-cold buffer C (20 mM HEPES [pH 7.9], 420 mM NaCl, 1.5 mM MgCl_2_, 20% [v/v] glycerol, 0.2 mM EDTA, 0.5 mM DTT, and 0.2 mM PMSF), incubated at 4°C for 20 minutes and centrifuged for 6 minutes at 14,000 *g*. Supernatant was collected as nuclear extract (NE). Protein concentration of NE was determined using a BCA kit (Bio-Rad, Richmond, CA).

### Electrophoretic mobility shift assays (EMSA)

EMSA was carried out in 25 μl of binding buffer (10 mM Tris [pH 7.5], 100 mM NaCl, 1 mM DTT, 1 mM EDTA, 4% [w/v] glycerol, 0.1 mg/ml sonicated salmon sperm DNA), using 10 μg of NE. Oligonucleotides containing consensus NF-κB (Promega, Madison, WI), MYC/MAX or p53 DNA recognition and binding sites (Santa Cruz Biotechnology, Santa Cruz, CA) were end-labeled to a specific activity of 10^5^ CPM, using γ-[^32^P]-ATP and T4-polynucleotide kinase followed by purification on a Nick column (GE Healthcare, Piscataway, NJ). Reaction mixtures were incubated at room temperature for 20 minutes and resolved on 6% non-denaturing polyacrylamide gels. Gels were dried and subjected to autoradiography. For super-shift assays, 2 μg antibody was added (20 min, ambient temperature) after the reaction with radiolabeled oligonucleotide had been completed. Antibodies to p50 (sc-114X), p65 (sc-109X), c-Rel (sc-70X), p52 (sc-298X), RelB (sc-48366X) or Myc (sc-764X) were purchased from Santa Cruz Biotechnology (Santa Cruz, CA).

### Microarray-based gene expression profiling (GEP)

GEP using the Affymetrix U133Plus 2.0 microarray (Santa Clara, CA) were performed as previously described [39]. Microarray data and outcome data on the 351 patients included in this study are available in the NIH Gene Expression Omnibus (GEO) under accession number GSE2658. Microarray data on the 44 individuals with MGUS and 22 samples of normal plasma cells (NPC) included here are available at GSE5900. Plasma-cell isolation, total RNA extraction, cRNA synthesis, and hybridization to microarrays were performed as described previously [18]. Statistical analysis of microarray data took advantage of the GCOS1.1 software (Affymetrix, Santa Clara, CA) and involved log-rank tests for univariate association with disease-related survival.

## Additional file

Additional file 1: Figure S1. Kaplan-Meier curves showing overall survival of DLBCL patients according to high or low expression of *CDKN1A* (202284_s_at). Data were mined from Dave et al. (GSE4732) [15]. Vertical hash marks represent a live patient at the indicated follow-up time. Mantel-Cox log-rank analysis was used to compare patient groups (not significant). Survival was recorded for 51 patients according to high and low p21 expression at diagnosis (left). Survival was also plotted for patients treated with either CHOP or a more intensive (INT) regimen (right).

## Abbreviations

BL: Burkitt lymphoma
EBV: Epstein Barr virus
EFS: Event-free survival
EMSA: Electrophoretic mobility shift assay
MM: Multiple myeloma
OS: Overall survival
PL: Piperlongumine
