# Supplementary figures and images for: CDKN1A and FANCD2 are potential oncotargets in Burkitt lymphoma and multiple myeloma
Source: Exp Hematol Oncol. 2015 Mar 27;4:9. doi: 10.1186/s40164-015-0005-2 (PMC4383050; doi:10.1186/s40164-015-0005-2)

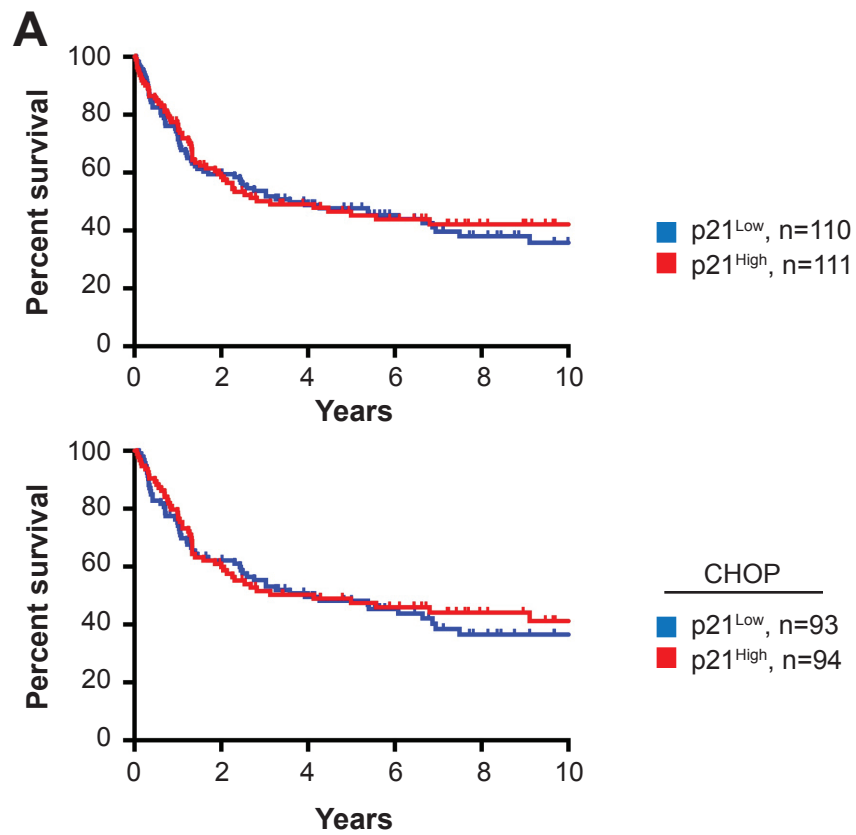

Supplemental Figure 1: Han *et al.*

Supplement: Additional file 1: Figure S1. — Kaplan-Meier curves showing overall survival of DLBCL patients according to high or low expression of CDKN1A (202284_s_at). Data were mined from Dave et al. (GSE4732) [15]. Vertical hash marks represent a live patient at the indicated follow-up time. Mantel-Cox log-rank analysis was used to compare patient groups (not significant). Survival was recorded for 51 patients according to high and low p21 expression at diagnosis (left). Survival was also plotted for patients treated with either CHOP or a more intensive (INT) regimen (right). [file 40164_2015_5_MOESM1_ESM.pdf]
